# “Data Free Flow with Trust”: Japan’s struggle to integrate democracy and human rights into digital trade policy

**DOI:** 10.3389/fsoc.2024.1397528

**Published:** 2024-09-25

**Authors:** John G. Dale, Nobuhiro Aizawa

**Affiliations:** ^1^Department of Sociology and Anthropology, College of Humanities and Social Sciences, George Mason University, Fairfax, VA, United States; ^2^Department of Cultural Studies, Graduate School of Integrated Sciences for Global Society, Kyushu University, Fukuoka, Japan

**Keywords:** digital transformation, Society 5.0, Data Free Flow with Trust, digital trade, data localization, Japan, G-7, human rights

## Abstract

A powerful regime for regulating trade, the Group of Seven (G-7) has increasingly negotiated its digital trade through bilateral and preferential trade agreements, including with non-member states in the Global South. Focusing on the dominant concept shaping these agreements, Japan’s “Data Free Flow with Trust” (D.F.F.T.), we trace its discursively contested emergence and meaning within a national (“Society 5.0”) vision for Japan’s digital transformation, and its subsequent transnationalization in international fora and institutionalization in global digital trade policy. Drawing on our interviews with Japanese government ministers, business elites, and legal experts who contributed to the processual development of D.F.F.T., as well as diverse additional primary sources, we find that the D.F.F.T. has become more than a trade policy, covering a wider range of social and geopolitical issues. In particular, we show that contention over “data localization measures” has restructured international relations of trust, especially across the Global North/South divide. Ultimately, this research report contributes to our understanding of how D.F.F.T. poses threats to human rights, democracy, and the global knowledge economy that may undermine its goals of enhancing innovation capacity and economic growth.

## Introduction

The world’s attention concerning the digital transformation of society is focused on the need for greater government regulation of generative Artificial Intelligence (AI), particularly on the competing visions and legislation of the European Union (EU), China, and the United States (see, e.g., Bradford, 2023; Hutson, 2023; Roberts et al., 2023). Bradford (2023, p. 7) argues, “These three leading regulatory models could be thought of as representing three “varieties of digital capitalism”—drawing on different theories about the relationship between markets, the state, and individual and collective rights,” whereby, “…the U.S. has pioneered a largely *market-driven* model, China a *state-driven* model, and the EU a *rights-driven* model”.

Insufficient attention, however, has focused on the rules and regulations that the G-7 currently is writing into the bilateral trade agreements with their partners in the Global South. A new generation of bilateral and preferential trade agreements between nation-states around the world have begun to include digital trade clauses reflecting different initiatives and strategies to regulate digital trade (Elsig and Klotz, 2021). For example, the United States-Mexico-Canada Agreement (USMCA) rules out local storage requirements (i.e., preempts claims to “data sovereignty”) to allow the “free flow of data,” while the European Union unilaterally exports and enforces law worldwide through its data protection laws.

Japan’s original concept of “Data Free Flow with Trust” (D.F.F.T.) more recently has been embraced by the G-7, which includes the United States and European Union. D.F.F.T. now shapes their emerging transnational digital trade policy discourse (Ministry of Internal Affairs and Communications, 2023). This marks a significant turning point for the institutionalization of global digital trade. Because the D.F.F.T. concept has now made its way into the G-7’s member states’ bilateral and preferential trade agreements between nation-states around the world, including those in the Global South, it is important to understand how it has influenced these agreements.

Critics and even some trade partners in the Global South have characterized the United States’ discourse on the free global flow of data as one justifying the legal status quo of “whoever collects the data owns it,” and Japan’s addition (“with trust”) as mere “window-dressing”(Singh and Vipra, 2019) to temper accruing resistance to an essentially neoliberal digital trade policy that ultimately benefits already dominant data market participants. Others have suggested that Japan’s policy concept brings to the G-7 debates a significant emphasis on personal privacy protection that has proved compatible with the EU’s General Data Protection Regulation (Wang, 2020). While G-7 member states typically distinguish their digital policies and practices as a democratic alternative to the “digital authoritarian” policies and practices of China (see, e.g., United States Senate Foreign Relations Committee, 2020), labor unions, civil society organizations, and academics have challenged the G-7’s practices for threatening democracy and human rights locally, transnationally, and globally (see, e.g., American Federation of Labor and Congress of Industrial Organizations (AFL-CIO) Technology Institute, 2023; Mumford, 2022; Transnational Institute et al., 2021; Singh and Joshi, 2023; and Couldry and Mejias, 2019). Our research suggests that the concept of D.F.F.T. has a complex history missing from current debates, and one which challenges its current depictions by both critics and supporters.

This Report begins by telling the story of the emergence of D.F.F.T., which was derived from the concept of “Society 5.0” coined in *The 5th Basic Law for Science and Technology* (*Basic Law*) and introduced in 2016. It traces the initially contested understanding of D.F.F.T. within a broader domestic struggle over Japan’s Society 5.0 vision to incorporate democratic and human rights principles into the digital transformation of its economy and society. Specifically, it shows how the meaning of D.F.F.T. was produced and transformed under conditions of domestic political contention over the implementation of Society 5.0.

The Report then examines how D.F.F.T took on new significance (and meaning) as Japanese Prime Minister Abe, in 2019, transposed the concept to an international context, specifically the World Economic Forum held in Davos, Switzerland and the G-20 Summit in Osaka. It further traces the concept’s elaboration under Japan’s subsequent Prime Minister Kishida when Japan held the Presidency of the G-7 and hosted the Ministerial Meetings at the Hiroshima Summit in 2023, where member states (especially the United States and EU) embraced D.F.F.T. as a guiding set of principles and justificatory discourse for expanding their digital trade policies. It shows that, in practice, one of the more contentious discursive formations animating new trade agreements based on D.F.F.T.—particularly those forged between G-7 member states’ and their trade partners in the Global South—is one that legally forbids practices of “data localization.” The Report also reviews some of the recent counter-discourses of labor unions, civil society organizations, and academics working across the Global North/South divide that challenge the democratic legitimacy of such trade clauses, and that identify significant human rights issues concerning the control and ownership of data.

The Report concludes by discussing two key shortcomings of the G-7’s efforts to integrate democratic and human rights principles into transnational digital policy, both of which may serve to undermine the economic innovation and growth that their policy purportedly intends to facilitate.

## Data and methods

The Report draws from intensive, semi-structured interviews ranging from 1 to 2 h in length (in addition to follow-up correspondence) conducted from October through December, 2021, with high-ranking officers in Japan’s Ministry of Economy, Trade, and Industry (METI) in the Robotics Policy Office, Manufacturing Industries Bureau, Digital Economy Division, and Commerce and Information Policy Bureau on its policy-planning process, as well as with business leaders in AI and robotics technologies in Japan, and business associations such as the Robotics Manufacturing Association and Keidanren. Series of interviews were also conducted with legal experts at University of Tokyo and Kyoto University who led the discussion on redefining human rights and governance under the digital transformation policy in Japan. We have collected primary information from white papers and policy papers, parliamentary session records and bi-lateral trade agreements, labor unions’ and non-governmental organizations technical reports and others for our analysis.

To examine the institutional discursive contestation over the vision for Society 5.0 (and the concept of D.F.F.T. that emerged from this contestation) *within* Japan’s ministries and *between* those ministries and powerful business associations and prominent Japanese constitutional legal experts, we conducted a transnational legal ethnography (Dale, 2011, pp. x–xiii and 24–26). This is a form of institutional ethnography that takes an archeological approach to representation (Burawoy, 1999, p. 65), which entails contextualizing local experiments and new institutional forms and translating them into a common language that links them to one another across the globe. However, an ethnography of transnational legal space represents not only an excavation of new legal terrains but also, as Dale (2011, p. xii) explains, “one of ‘globalized’ territory, i.e., a space of hegemonic discursive flows that threatens to strip mine such new legal terrains of the opportunities they provide for debating, confronting, and negotiating political, legal, and moral norms and values. It is an interpretive project to understand a temporally unfolding location of relations among contingent and conflicting representations before their discursive resolution within the fixed boundaries of any particular institutionalized outcome”.

## Results

### The national emergence and transnationalization of “digital free flow with trust”

Appreciation for why the United States and European Union—each holding disparate views on data trade policy—would mutually embrace Japan’s concept of D.F.F.T. begins with understanding the national context in which the concept emerged. In 2016, the Japanese Government publicly introduced its national vision for the type of society they sought to produce by fundamentally integrating digital technology into its existing political economy. “Society 5.0,” as they called it, was defined as “a human-centered society that balances economic advancement with the resolution of social problems through a system that highly integrates cyberspace and physical space” (Cabinet Office of Japan, 2016a). This was designed to be a model that produces “a high degree of convergence between cyberspace (virtual space) and physical space (real space),” and “a new society that incorporates these new technologies in all industries and social activities and achieves both economic development and solutions to social problems in parallel” (Cabinet Office of Japan, 2016a). In short, Society 5.0 was a national vision to establish a human-centered society in a digitalized era.

More specifically, this idea was originally to establish a model for a democratic and sustainable relationship between society and the state—one consistent with international human rights norms and the UN’s sustainable development goals—that could withstand anticipated efforts to automate work and governance to solve Japan’s major social challenges such as an aging demographic structure and natural disaster recovery. It was envisioned as an alternative to China’s “surveillance state” and the U.S. Silicon Valley’s “surveillance capital” models for the development of a global knowledge economy. Unlike China and the United States, Japan has few home-grown global corporate technology platforms. Japan now stands at a critical economic juncture: emerging from a two-decade period of economic stagnation, it seeks a way to secure national autonomy and boost its innovative capacity and competitiveness to prosper in this rapidly changing knowledge economy.

Evidence-based policymaking in Japan largely has been the exclusive domain of the natural and physical sciences, technology, engineering and mathematics. However, this political agenda emphasized the need for integrating the social sciences and humanities (including law) into discussion of the ethical, legal and social issues relevant to implementing new science and technology. In 2016, Japan formally amended its *Basic Law* (Cabinet Office of Japan, 2016b, p. 47) to clarify that it was intended to help citizens understand the social costs and benefits of embarking on this new vision, and to forecast unexpected perils and set preventive measures. The Basic Law encouraged technological advancement and ongoing scientific assessment of the impact of these emerging technologies on Japan’s society.

But the purpose of building a human-centric society through digital technological development was not free of domestic political contention. Three primary sets of actors sought to shape how, and toward what end, this digital transformation would unfold: (1) the Prime Minister’s Office, which promoted the concept as a solution for addressing social problems (such as an aging society, economic disparity, and rural depopulation), and as a guiding principle for achieving a sustainable “human-centric” society through digital technology; (2) The Ministry of Economy, Trade, and Industry (METI), along with Keidanren (the Japanese Business Federation) and major business groups who persistently emphasized the pressing need for prioritizing economic development through technological advancement in a time when Japan is struggling to meet its growth benchmarks; and (3) legal academics, some of whom had worked stints in the Ministry of Justice, who emphasized the need for enhancing Constitutionally rooted privacy rights and—as both a basic democratic principle and as a human right—public access to information.

Within this contested domestic politico-legal space from 2016 to 2018, two distinct but mutually influential policy strategies emerged. The first strategy was to tie the Japanese economy to the U.S.’s most advanced practices of capitalism in the knowledge economy. There was a major push by Japan’s business stakeholders to build upon the existing liberal economic development model. For decades, Japan has been suffering low population growth due to its aging society. Additionally, METI analysts were forecasting disruptive factors to economic growth, such as the energy and food crises. An agreement over economic development as the path toward Society 5.0 soon gained government support. In order to reap the economic benefits of a growing global digital technology industry, Japan chose to navigate the growing tension between the U.S. and China’s technological development by working with U.S. tech companies such as Amazon and Google to make the U.S.’s technological ecosystem accessible to Japanese business and government (Suzuki, 2021).

The second strategy was to forge a legal alliance with the EU. By 2018, legal experts in Japan saw the EU’s General Data Protection Regulation (GDPR) as an opportunity to form an alliance with European states to address the foreseeable concerns over rapidly eroding privacy rights associated with U.S. Big Tech firms (Zuboff, 2019; Cohen, 2019), and uphold the Japanese Constitution’s strict privacy rights, such as the privacy of communication, vis-a-vis both the growing global tech giants and the growing power of the state. These concerns were already being discussed among Japan’s legal experts in terms of data privacy for basic principles of Society 5.0, but by 2019 they would contribute to an Amended Act on the Protection of Personal Information^1^ and a landmark GDPR Adequacy Agreement with the EU (Wang, 2020; Commission Implementing Decision (EU), 2019).

By 2019, Japan had come to define the “trust” in D.F.F.T. in terms of four principles: privacy, security, intellectual rights and data protection. When Japan enunciated these principles, first in Davos and then in the summit meeting of G-20, it presented them largely as a general and overarching idea. In his January 2019 address to the World Economic Forum in Davos, Prime Minister Abe proclaimed that “the engine for growth” is “fueled no longer by gasoline, but more and more by digital data” and called for the international community to build a global infrastructure grounded in the principles of Society 5.0—particularly its economic and legal principles, or now, D.F.F.T.:

The regime we must build is one for D.F.F.T., Data Free Flow with Trust—non-personal data, needless to say. It is not the big, capital intensive industries, but rather we individuals who will benefit from both the fourth industrial revolution and what we call ‘Society 5.0,’ which this fourth industrial revolution will bring about.^2^

Five months later, at the G-20 Summit, which included China and Russia, he introduced the launch of an “Osaka Track”:

…a process that aims to promote rule-making under the “Data Free Flow with Trust” concept. We will speedily advance international rule-making to ensure cross-border data free flow while protecting privacy and security. This will no doubt breathe new life into the WTO reform process (Prime Minister of Japan and His Cabinet, 2019).

The G-20 agreement was based more on D.F.F.T. as an “idea” than a “policy,” and any resulting policy would be left in the hands of countries who shared the idea. Keita Nishiyama, the *de facto* architect of the DFFT concept, and former Director General of the Commerce and Information Policy Bureau at METI, stated that, with this approach, “the strategic intention was an ambitious one to bridge not only the US and the EU, but also to create a common policy platform that the U.S. and China could both accept. It was meant to establish a new standard of political legitimacy over digital trade rules, a concept of “trust” that could be used across different political regimes with wide-ranging stances on cross-border data regulation.”^3^

The substantive policy-making, in 2019, was to take place within the World Trade Organization (WTO), which was mandated at the G-20 meeting as “Osaka Track.” The discussion at the WTO was framed under the rubric of E-commerce, and Japan, together with Australia and Singapore served the role of co-convenors to lead the process of forging a WTO joint statement initiative (Fukunaga, 2019; Australia, Japan, Singapore, 2023). This proved to be a pivotal moment in the institutionalization of D.F.F.T. as a trade policy, in which the concept of “trust” was secured through trade rules. This clearly was a reset from Abe’s previous approach that took place in the Asia-Pacific Economic Consortium in 2015, where the principle of protecting personal data had little chance of garnering sufficient support for an agreement amidst simmering tensions with China, and clearly sharpening differences between the US and EU, particularly with respect to repeated challenges concerning the EU-US Privacy Shield (a mechanism to facilitate compliance with EU data protection requirements when transferring personal data from the European Union to the United States in support of transatlantic commerce).

### Re-locating the boundaries of “trust”

Japan’s approach in 2019 was to use international fora to frame global principles for addressing both domestic and international concerns. Based on business analyses it received from Keidanren, Japan then saw value in courting China as well as the U.S. as digital trade partners. But this strategic approach of convening all of its potential partners was soon abandoned. With the COVID crisis of 2020–2022, the elevated tensions between the US and China, and the geopolitical challenge posed by Russia’s invasion of Ukraine in 2022, Japan gave up on efforts to find a mutually acceptable policy platform for the two superpowers, and instead prioritized its relationship with the G-7 members and foregrounded a new geopolitical principle in framing the D.F.F.T concept.

In May 2022, Japan’s new Prime Minister Kishida, gave a keynote speech to investors in the City of London elaborating the concept of D.F.F.T. It raised concerns over global inequality that might stem from an unchecked free flow of data, and shed light on who is (and who is not) to be trusted:

I would like to introduce to you an economic policy I am advocating which I call [a] ‘new form of capitalism.’ … Japan will grow by being connected to the rest of the world through the free movement of people, goods, money, and digital technologies across borders. …Why does capitalism need an upgrade? Because we need to solve two present- day challenges. One is the problem of economic externalities, such as widening inequality, climate change and issues deriving from urbanization. …The second pressing challenge is that posed by authoritarian states. Liberalism and democracy are under pressure from authoritarian regimes. We must make economies in democratic nations sustainable and inclusive in order to defend freedom and democracy.^4^

In 2023, Japan occupied the Presidency of the G-7 and hosted the Ministerial Meetings at the Hiroshima Summit, where discussions over how to regulate generative AI were highly anticipated, as the EU and China were already preparing to announce in coming months new AI laws. The day before the Summit, Prime Minister Kishida published an essay in *Foreign Affairs*, “The New Meaning of Hiroshima: At Japan’s G-7 Summit, We Must Both Defend Global Order and Address Global rises.” In his essay, Kishida announces, “The world is at a historic crossroads. It is facing a complex of crises…,“and then highlights (in addition to climate change, pandemics, food and energy insecurity) two geopolitical crises. The first geopolitical crisis he describes this way:

…Russia’s aggression against Ukraine, which has shaken the very foundations of the international order. At the G-7 Hiroshima summit, held against this backdrop, we must powerfully demonstrate our determination to uphold a free and open international order based on the rule of law. At the same time, we must also strengthen our outreach to the countries of the so-called global South. Russia’s aggression against Ukraine has had a devastating impact on people’s livelihoods across the world, but especially in the global South. Unless we listen to and address the concerns related to that impact, we will fail to build the *trust* necessary to uphold a free and open order (Kishida, 2023, emphasis added).

Here, Kishida ties “the trust” in D.F.F.T. to global inequality, but particularly as it has been impacted by Russia’s aggressive attack on a “free and open international order based on the rule of law.”

The second geopolitical crisis he associates with China’s militant action in the Indo-Pacific region, which threatens peace and security, and calls for cooperative national defense among G-7 “allies and like-minded partners” to constructively engage China:

In Hiroshima, G-7 leaders will deepen our discussions of the Indo-Pacific so that the G-7 is aligned in responding to regional challenges. China’s current external stance and military activities are a matter of serious concern to both Japan and the international community and present an unprecedented strategic challenge to peace and stability. This challenge must be addressed through robust national defense and cooperation among allies and like-minded partners, as well as through regular dialogue with China aimed at building constructive and stable relations.

It was at the G-7 Hiroshima Summit that member states (including the U.S. and EU) publicly embraced D.F.F.T. as a guiding set of principles and justificatory discourse for expanding their digital trade policies.^5^ In this process, the D.F.F.T. has become not only a trade policy but an industrial, social, and security policy, covering a wider range of social and geopolitical issues, and emphasizing trust more than the free flow of data.

## Discussion

New rules and regulations for digital trade are now being written through regional and bi-lateral economic partnership agreements between states around the world. Japan’s concept of D.F.F.T. has had significant influence, not only among G-7 member states and the their partners in the Global South (Information Technology and Innovation Foundation and Cory, 2023), but also within other regional trade associations like the Association of Southeast Asian Nations (ASEAN), the Regional Comprehensive Economic Partnership (RCEP), Comprehensive and Progressive Agreement for Trans-Pacific Partnership (CPTPP) as well as the Indo-Pacific Economic Framework for Prosperity (IPEF). This marks a significant turning point for the institutionalization of global digital trade. But, as our results show, this process was far from what Japan had originally intended for its own digital transformation.

Japan’s strategic choice of using the concept of trust in the form of D.F.F.T. as a bridging concept and policy platform has created a space within multiple and diverse economic bodies for discussion between the U.S., EU, and China that was untenable just a few years ago. But it also unintendedly has concentrated attention to, and exacerbated contention over, the issue of “data localization measures” and relatedly, data ownership and control.

Data localization measures can be roughly defined as “laws and regulations that restrict or enclose the cross-border transfer of data” (Yoshinori, 2021). All the members of the G-7 include in their trade agreements with partners in the Global South (and with each other) prohibitions on data localization and processing. Of course, in practice, they themselves do engage in various forms of data localization, setting limits on the complete “free flow” of data. Disagreements focus on the type and extent of data localization measures. Narrower measures may require actors to domestically store data related to their local activities, or install data processing servers domestically, as in the case of China’s Cybersecurity Law.^6^ Broader measures may require actors to regulate the cross-border transfer of such data in order to protect the privacy and personal information of the public. The EU’s GDPR, for example, regulates such cross-border data transfer, but it does not impose domestic data storage or facility installation requirements. And at their 2023 Higher Level Economic Dialogue, the EU and Japan agreed a cross-border trade deal to remove domestic data storage or facility installation requirements.^7^ But as Yoshinori has observed, data-localization provisions can be drafted ambiguously on purpose—an act he calls “constructive ambiguity”—in order to obtain the agreement of the negotiating countries, leaving the details to be clarified or disputed through subsequent processes (Yoshinori, 2021, p. 26).

In 2016, the United Nations Conference on Trade and Development expressed concern for this approach to securing data flow regulation, stating, “…[I]nternational trade agreements are often seen as being developed through secretive negotiations that appear to severely limit opportunities for a consumer/civil society voice to be heard. …The development of global and regional data protection initiatives also requires engagement with developing nations. Too often the debate is dominated by the interests of developed nations” (United Nations Conference on Trade and Development (UNCTAD), 2016, p. 63). Recognizing the outsized influence of Big Tech companies shaping such trade agreements, the UN Human Rights Council has since established the B-Tech project to prevent, remedy, and address human rights harms related to digital technologies, applying the *UN Guiding Principles on Business and Human Rights* to the design, development and use of digital technologies. This concern is echoed in the UN Committee on Economic, Social, and Cultural Rights’ General Comment No. 25 on a human right to science advising that “States parties should establish a legal framework that imposes on non-State actors a duty of human rights due diligence, especially in the case of big technology companies. …Moreover, taking into account that many of the emerging inequalities are strongly linked to the capacity of some business entities to access, store and exploit massive data, it is crucial to regulate the ownership and control of data according to human rights principles” (United Nations Committee on Economic, Social and Cultural Rights (UNCESCR), 2020, at ¶ 75–76).

Data localization measures being written into the digital trade clauses of economic partnership agreements have raised concerns among observers in the Global South. Singh and Vipra (2019, p. 54), for example, argue that, “…once countries give up their right to check data from freely flowing out, no data ownership rules—or other kinds of economic rights over data—can ever be meaningfully instituted by them. That would be the end of any chance of a digital economy which is fair to small actors and to developing countries”. They point out that, although the D.F.F.T. principles claim to address the concerns of developing countries by including privacy and security protections in their frameworks, they nevertheless skirt the more important issue that these countries voice about domestic economic rights over data flowing from their shores. An OECD analysis of existing measures reveals that, in 2021, there were total of 92 data localization measures in place across 39 countries—and that data localization is on the rise. Importantly, these measures have become more restrictive: by 2021, two-thirds of measures in place involved a storage requirement with a flow prohibition—often implemented by non-OECD countries (Gonzalez et al., 2022).

As Singh and Vipra note, at issue is not the intellectual property rights of those collecting data, but rather “…the rights of people, communities and small economic actors that contribute the data and whom the data is about” (Singh and Vipra, 2019, p. 55). What the G-7 (and western Big Tech companies) call “data localization” and “digital (economic) protectionism,” many states, indigenous nations, and trans-local networks of municipalities in the Global South call “foreign data extraction” or “data colonization” and “digital sovereignty,” and they are working to build alternative futures for their own digital development. For example, the Maasai People’s Data Project in Tanzania is a community-led initiative that aims to protect the cultural heritage and land rights of the Maasai people through the creation of a sovereign data infrastructure (Basu and Sinha, 2023). The Maori Data Sovereignty Network in New Zealand is a group of indigenous researchers, practitioners, and activists are asserting Maori rights and interests over Maori data and developing indigenous-led frameworks and protocols for data governance and stewardship (Walter, 2021). And the Regional Agreement on Access to Information, Public Participation and Justice in Environmental Matters (the “Escazú Agreement”) entered into force in late 2020 and ensures every city among all the Latin American signatories releases its specific environmental open datasets, receiving investment in municipal resources for generating, collecting, publicizing, and disseminating environmental data. As Avila and Weress (2023, p. 18) observe, “The Escazú Agreement could be a solid base for creating a federated commons of environmental data” and “provide a legal basis for a practical blueprint for all cities, an ‘environmental information’ data commons for climate action, built from general norms and a regional commitment in the Global South”.

While the 2023 EU-Japan agreement addresses mandated access to digital data held by private companies that is needed for public interest purposes,^8^ there is no discussion among G-7 countries of alternatives to unified property rights that would enable differently structuring economic rights to data (Unger, 2019; See also Keller and Block, 2023). Brazilian philosopher, jurist, and politician Roberto Unger argues,

A special problem and unique opportunity exist with respect to the part of the knowledge economy that trades in the data of millions of people. …Data should belong to the individuals who generate them, as part of the expression of personality in society. Those who use data for economic gain should win consent for their use and pay for them. The radical decentralization of property in data… would encourage a wide range of varieties of compensation other than the payment of a rent by the data user to the data generator. Such alternative variants of remuneration would include fractional equity stakes. …The result would be to turn passive sources of material into engaged agents (Unger, 2019, pp. 128–30).

There are currently a number of creative efforts underway around the world to develop collectively owned, democratically governed data commons and digital platform cooperatives (Bühler et al., 2023),^9^ which would find little conceptual, much less legal, space for legitimate consideration within the digital transformation envisioned by these new digital trade agreements. This suggests a serious shortcoming of the D.F.F.T. principles—and of Japan’s original “human-centric” vision of “Society 5.0.” They focus too narrowly on individual economic (human) rights to the exclusion of collective economic (human) rights.

There is another shortcoming of D.F.F.T. that relates to the democratic aspirations Japan once envisioned for Society 5.0. As D.F.F.T has come to influence the institutionalization of particular (narrow) arrangements of data localization, with its stated justification of defending against potential local authoritarian use of data (presumed to occur outside of G-7 member states and disproportionately within the Global South), it also risks stymieing potential local democratic uses of data and the development of legitimate local business enterprise and competition both in the Global South as well as among market participants in Japan who might hope to organize collective data ownership through data commons and platform cooperatives. This suggests one way that D.F.F.T. contributes to a structural, competitive disadvantage within the knowledge economy for domestic market participants in the Global South. It highlights an asymmetrical relation of power, forged in part through a form of “trust” constructed via rules shaping data localization, that likely will exacerbate the already existing global inequality within the knowledge economy.

Taken together, these shortcomings point to a fundamental flaw in the underlying assumption driving the dominant D.F.F.T. digital trade policy discourse. The G-7’s consistently articulated goal in embracing D.F.F.T. has been to unleash data in ways that contribute to greater capacity for innovation and, ultimately, sustainable economic growth. China too has vociferously embraced this goal. Yet, by concentrating control of data largely to the already most powerful firms through data localization bans that unequally empower their data collection practices, and by confining opportunities to expand and experiment with new collective (private and public) forms of data rights and ownership, D.F.F.T. has suffered a failure of vision. It has more likely set a course for diminishing global innovative capacity and productivity, de-democratizing knowledge production, and exacerbating already unsustainable levels of social and economic inequality.

## Data Availability

The raw data supporting the conclusions of this article will be made available by the authors, without undue reservation.

## Footnotes

^1^The Amended Act on the Protection of Personal Information (approved in 2020 and enacted in 2021) is available from: https://www.ppc.go.jp/files/pdf/280222_amendedlaw.pdf (Accessed on 29 January, 2024).

^2^English translation of Abe’s speech is available at https://www.weforum.org/agenda/2019/01/abe-speech-transcript/ (Accessed on January 17, 2024).

^3^Interview with Nishiyama on November 18, 2020.

^4^English translation of Kishida’s speech is available at https://www.japan.go.jp/kizuna/2022/06/why_japan_is_a_buy.html (Accessed on January 17, 2024).

^5^See “G-7 Hiroshima Leaders’ Communiqué,” (May 20, 2023), especially at Para. 39. Available from: https://www.whitehouse.gov/briefing-room/statements-releases/2023/05/20/G-7-hiroshima-leaders-communique/ (Accessed 31 January, 2024).

^6^Available from: https://digichina.stanford.edu/work/translation-cybersecurity-law-of-the-peoples-republic-of-china-effective-june-1-2017/ (Accessed 5 February, 2024).

^7^Available from: https://policy.trade.ec.europa.eu/news/eu-and-japan-start-negotiations-include-rules-cross-border-data-flows-their-economic-partnership-2022-10-07_en (Accessed 5 February, 2024).

^8^The AFL-CIO Trade Institute, in January 2023, called upon the Biden Administration to “authorize governments to enact data localization policies with regard to certain categories of sensitive data.” They focus specifically on keeping onshore individuals’ sensitive personal information and data related to certain sectors (such as critical infrastructure, national security, and law enforcement) to ensure it is subject to enforceable privacy standards and effective government oversight. But much of this could be addressed by the EU-Japan agreement’s mandated access for public interest purposes.

^9^See also, Thiruvananthapuram Declaration on A New Innovation Ecosystem for Our Collective Digital Futures. Available from: https://platform.coop/blog/the-thiruvananthapuram-declaration-on-a-new-innovation-ecosystem-for-our-collective-digital-futures-2/ (Accessed on: 5 February, 2024).
